# Surface Finishing of Additive Manufactured Ti-6Al-4V Alloy: A Comparison between Abrasive Fluidized Bed and Laser Finishing

**DOI:** 10.3390/ma14185366

**Published:** 2021-09-17

**Authors:** Eleonora Atzeni, Silvio Genna, Erica Menna, Gianluca Rubino, Alessandro Salmi, Federica Trovalusci

**Affiliations:** 1 Department of Management and Production Engineering, Politecnico di Torino, Corso Duca Degli Abruzzi 24, 10129 Torino, Italy; eleonora.atzeni@polito.it (E.A.); alessandro.salmi@polito.it (A.S.); 2 Department of Enterprise Engineering, Università degli Studi di Roma “Tor Vergata”, Via del Politecnico 1, 00133 Roma, Italy; silvio.genna@uniroma2.it (S.G.); federica.trovalusci@uniroma2.it (F.T.); 3 Department of Economics, Engineering, Society and Business Organization, Largo dell’Università, Università della Tuscia, 01100 Viterbo, Italy; gianluca.rubino@unitus.it

**Keywords:** laser finishing, fluidized bed, surface roughness, fatigue, EBM, Ti-6Al-4V

## Abstract

Metal additive manufacturing is a major concern for advanced manufacturing industries thanks to its ability to manufacture complex-shaped parts in materials that are difficult to machine using conventional methods. Nowadays, it is increasingly being used in the industrial manufacturing of titanium-alloy components for aerospace and medical industries; however, the main weakness of structural parts is the fatigue life, which is affected by surface quality, meaning the micro-cracking of small surface defects induced by the manufacturing process. Laser finishing and Abrasive Fluidized Bed are proposed by the authors since they represent cost-effective and environment-friendly alternatives for automated surface finishing. A comparison between these two finishing technologies was established and discussed. Experimental tests investigated both mechanical properties and fatigue performances. The tests also focused on understanding the basic mechanisms involved in fatigue failures of machined Ti-6Al-4V components fabricated via Electron Beam Melting and the effects of operational parameters. X-ray tomography was used to evaluate the internal porosity to better explain the fatigue behaviour. The results demonstrated the capability of Laser finishing and Abrasive Fluidized Beds to improve failure performances. Life Cycle Analysis was additionally performed to verify the effectiveness of the proposed technologies in terms of environmental impact and resource consumption.

## 1. Introduction

Metal additive manufacturing (AM) is proving to be a powerful technique in response to an ever-increasing demand for customized and complex products, since it is promisingly becoming an important component of industrial manufacturing, not only for its use in modern rapid prototyping or small-series fabrication but also for large-scale production [1,2]. Components are built layer-by-layer using powder bed fusion (PBF) methods, without the need for expensive dies or complex equipment. These benefits become particularly attractive when related to high-strength alloys difficult to machine with conventional technologies [3,4]. Electron beam melting (EBM) is rapidly establishing in the field of metal AM technologies. Thanks to the vacuum environment and high build temperatures, EBM assures the production of fully dense parts with high quality and near net shape. Therefore, it is perfectly suitable to meet even the strict requirements of the high-strength titanium alloys used in the aerospace and medical industry [5,6,7,8], with as-deposited properties in the range of those of cast or wrought materials (and in some cases still greater) [9,10].

Titanium alloys are of particular interest, especially in the aerospace and biochemical sectors, because of their high strength-to-weight ratio, greater corrosion resistance, good biocompatibility, and extreme toughness. However, enabling the EBM machining of fatigue loading applications is still a goal to be secured [11]. Additively manufactured parts unavoidably have anisotropic properties and process-dependent microstructure related to building direction and specific thermal history of surface layers [12]. Spherical pores and lack-of-fusion (LoF) defects are typically induced during the EBM process [13]. The shallower the position of defects, the more detrimental to fatigue life effect [14,15]. Therefore, in acting as a stress concentrator on multiple sites, surface roughness is generally known to be primarily responsible for fatigue failures. Fatigue crack-initiation occurs mostly on the free surface where the constraint to cyclic sleep is lower [16]. 

The impact of these drawbacks may be successfully mitigated through optimization of process parameters. Partially remelting of the as-built material is technically necessary to reach proper interlayer metallurgical bonding and geometrical accuracy control. Vastola et al. performed a finite element method to model phase transitions in high-energy beam additive manufacturing through the interaction of beam parameters. It was found out that there exist optimal beam shape and size (which in turn depends on the scan speed) that correspond to the maximum remelt volume fraction [17]. Hrabe and Quinn investigated the effects of intra-build variations on microstructure and mechanical properties. It was reported that part size had a slight influence on mechanical properties, whereas the distance from the build plate did not affect [18]. Besides, the increase of energy input was associated with both the decrease of mechanical properties and the increase of α lath thickness and prior-β grain size [19]. Małecka and Rozumek studied the effects of phase composition. They found that the mechanism of cracking is affected by phase composition due to the difference in plasticity between the α phase and β phase [20].

The complexity of manufacturing process control and the substantial improvement of fatigue performance observed in response to the reduction of surface roughness encourage the machining of additively manufactured critical parts [21]. Therefore, surface quality is essential for leveraging the full potential of metal AM in the industrial environment, and, therefore, post-processing is a viable solution to scale up operations and comply with sustainability requirements at once. 

Mechanical machining such as milling, blasting, and grinding are industrial common processes for surface finishing of additively manufactured parts. Bagehorn et al. [22] carried out a comparative study on different mechanical finishing procedures aimed at evaluating their effects on fatigue performance. Milling proved effective in reducing surface roughness and improving fatigue behavior. However, the applicability of the milling process is limited by the complexity of the shape of the component to be machined. Vibratory grinding was additionally performed by the authors using a vibrating bowl filled with a fluid compound and small abrasive particles. It is stressed that such a process, as well as milling, is in itself time-consuming. Blasting was found to induce subsurface compressing stress which amends fatigue performances. In contrast, it takes a shorter processing time and involves smaller accessibility issues related to complex shapes. Nevertheless, the residual roughness should be removed through an additional fine polishing step. The same applies to micromachining. Ahmed et al. [23] achieved high surface finishing quality successfully combining additive and subtractive methods by rotary ultrasonic machining of additively manufactured parts. However, this technique requires long treatment times. Besides the results are sensitive to parameters setting. Electropolishing is commonly used to improve the surface finish of metals. However, contaminants and films interfering with metal dissolution are detrimental to electropolishing quality. Therefore, electropolishing requires pre-treating processes such as surface cleaning, surface modification, and rising. Process parameters have to be carefully tuned in order to optimize polishing conditions. Moreover, electrolytes commonly use perchloric acid which is dangerous and highly explosive [24]. Only a few perchlorate-free electrolytes are proposed containing sulphuric acid in substitution [25].

Among finishing processes, non-conventional technologies are particularly suitable for improving surface finishing of additive manufactured parts, since they avoid tool wear and physical damage arising from heavy mechanical stresses of the smallest components, and being the tool non-massive, they also allow the machining of more complex geometries. In particular, Abrasive fluidized bed (AFB) and laser finishing (LF) stand out for resource consumption and waste reduction. In the present study, they were both investigated as potential candidates for improving the surface finishing and fatigue behavior of Ti-alloy components fabricated via EBM. 

LF is based on selective surface modification through remelting of a thin surface layer and subsequent solidification under surface tension [26]; whilst in AFB, surface finishing is established by repeated impacts of abrasive media [27]. Both of these methods ensure low operating costs and no waste problems. Emissions in the environment are at near ambient conditions and free of lubricants or chemicals at any stage of the process. Moreover, they are perfectly suitable for automated surface finishing of complex-shaped parts.

Based on preliminary investigations of the effect of process parameters on fatigue performances [28,29], the study provides the description of the main mechanisms causing fatigue failure. Computer tomography (CT) scanning and image analysis were used to evaluate the internal porosity of EBM parts. A comparative life cycle assessment (LCA) was additionally carried out to ensure minimal environmental impact. The aim is to establish a comparison between these two finishing technologies to take full account of environmental issues while improving the fatigue life of additively manufactured Ti-6Al-4V alloy to fully exploit the strengths of metal AM.

## 2. Experimental Procedures

### 2.1. Material and Additive Manufacturing

**Figure 1:**
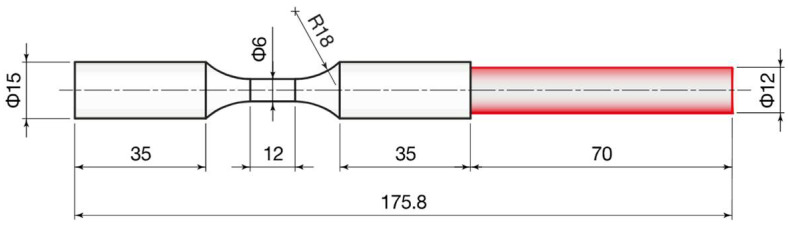
Geometrical features of samples, dimensions in mm.

The metal powder chosen for the experimentation was a gas-atomized grade 23 titanium alloy Ti6Al4V ELI (Fort Wayne Metals, Fort Wayne, IN, USA) whose particle size was in the range of 45–100 µm. The fabrication of samples was performed according to the EBM process using an Arcam A2X system (GE Additive, Norwalk, CT, USA) operating under vacuum. The main characteristics of the EBM system are listed in Table 1. The layer thickness was fixed at 50 µm. A pre-heated process chamber and high melting capacity of the EBM system avoid the arising of residual stresses and martensitic structures in the printed parts [30]. For further details on the printing procedure, reference is made to a previous work dedicated to finishing of additively manufactured Ti6Al4V parts [28,29]. EBM samples were built with an axial-symmetric geometry (Figure 1) typical of fatigue test specimens, in order to systematically investigate the influence of AFB and LF treatments on the fatigue life of parts. The yield stress of the material is 830 MPa, evaluated by performing static tensile tests in the as-build state [29].

### 2.2. Laser Processing

A diode-pumped ytterbium fiber laser (IPG YLR-450/4500-QCW-MM-AC-Y14, IPG Photonics, Milano, Italy) with an output power of 450 W operating at the wavelength of 1070 nm was used to finish the rough surface of EBM samples. Two main modes of laser emission are possible: continuous (named Continuous Wave, CW) and pulsed (also called Quasi-continuous wave, QCW) mode. The laser system is characterized by high-quality fiber output (M^2^ factor of 5.87), high power (maximum average power of 450 W and maximum peak power of 4500 W in QCW mode), reliable long lifetime, compactness, efficiency, and external computer interface. In the present study, CW mode was selected as it promotes surface smoothing. Nitrogen was used as assisting gas to prevent oxidation. Following laser finishing, samples were sandblasted with an abrasive pink corundum powder (mesh size 120) at a pressure of 4 bar to remove any incoherent material and then cleaned in an ultrasonic bath. The focus distance (Fd) was set at 5 and 7.5 mm, and for each focus condition, the scan speed (Ss) assumed the values 3.6, 9 and 18 mm/min.

### 2.3. Fluidized Bed Processing

The fluidized bed was composed by an air supply system and a vertical fluidization column, 200 mm in height and 500 mm in diameter [29]. The abrasive media in the bed was uniformly fluidized through a porous plate distributor by supplying a fluidizing gas (purified air). The abrasive particles were inert and there were no reactions inside the bed. Steel powder Type S, mesh size 12 μm, angular steel grit, 800 HRV was used as abrasive. Samples were rotated to ensure process uniformity on the whole surface. In particular, the cylindrical part of the samples was clamped on a horizontal shaft rotating at 4800 rpm and 6000 rpm, kept in the inner part of the fluidization column. Samples were exposed to the action of the abrasive for different treatment times: 5 h, 7.5 h and 10 h. Higher times were not considered because of the reduced industrial interest in longer processes. After finishing, samples were cleaned in an ultrasonic bath.

### 2.4. Characterization

In the middle of the smaller diameter portion of one sample, micro-CT scanning and image analysis were performed in order to evaluate the internal porosity. A phoenix v|tome|x s240 by GE Inspection Technologies (Boston, MA, USA) X-ray inspection system, equipped with a 240 kV/320 W microfocus tube, was used to scan the sample, held by a three-jaw chuck. The voxel resolution was 7.5 µm. The scan settings were 138 kV, 55 µA, with 0.5 mm thick Sn beam filter, and 1000 acquisitions were taken in a full 360° rotation. At each position, three images were acquired, of which the first one was discarded and the other two averaged. The data analysis was performed in VGSTUDIO MAX 3.4 by Volume Graphics (Heidelberg, Germany) software tool following the methodology proposed by du Plessis et al. (2018) [31], based on the direct segmentation method. The edges, that in AM typically present high roughness and open pores, were removed from the analysis by eroding the volume by 7 voxels (approximately 50 µm) and the threshold between material and pores was selected by picking the outer zone (left to the material histogram).

The morphology evolution of the titanium samples due to the AFB and LF treatments was analyzed by using a Leo SUPRA 35 FEG-SEM by Zeiss (Thornwood, NY, USA) and through the TalySurf CLI 200 contact gauge profilometer by Taylor Hobson (Leicester, United Kingdom). Three-dimensional maps were acquired by the profiler, adopting a Gaussian filter. All the 2D roughness parameters were evaluated for each working condition, three repetitions of the test were performed to calculate the average roughness and the standard deviation. According to the characterization procedure, the influence of the operational parameters set for the compared treatments (AFB and LF) on the surface morphology was assessed. Surface Hardness was also obtained for each treatment condition by a micro-scale tester (Micro-Combi, CSM Instruments, Peseaux, Switzerland), using a Vickers indenter.

Fatigue tests were performed by using the 2TM831 rotating bending machine by Italsigma (Forlì, Italy). The fatigue failure of as-built samples and after AFB and LF treatments was evaluated by adopting a rotating bending testing procedure. Once fixed the applied load, 7.8 kg, the number of cycles to failure was determined for each treatment condition, identifying the conditions beneficial to the fatigue life.

### 2.5. Life Cycle Analysis

An environmental impact assessment associated with the production of the specimens and the two finishing technologies was carried out. The LCA model was implemented with the SimaPro software (SimaPro 9.2, PRé Sustainability, Amersfoort, The Netherlands), starting from the production cycle. In this analysis, the authors did not only consider the environmental effects deriving from the production process, but also the raw materials supplying phase. The LCA method is regulated by the series ISO-14040 standards and provides for the definition of 4 main phases: Scope and objective definition; Inventory analysis; Impact estimate; Interpretation. Firstly, the components to be analyzed for the impact assessment were defined. The authors decided to analyze 1 sample for laser treatment and 4 samples for AFB, to have a comparable number of cycles to fatigue failure. In particular, in defining the LCA models associated with each finishing technologies, it was decided to consider the following flows:

- Material: raw material, support material, chemical agents, etc.;
- Energy: compressed air, electricity.

Regarding the production of the specimens, both the pre-manufacturing and EBM manufacturing steps were considered. The embodied energy to produce the Ti6Al4V powder was assumed to be 623.4 ± 5.6% MJ/kg. This value accounts for the primary production of the Ti6Al4V alloy, considers the use of 22% of recycling content and the energy for recycling, and includes the energy demand for gas atomization [32]. Powder atomization losses were quantified in 3% of the produced powder mass according to Paris et al., 2016 [33]. The EBM lasted 56.6 h, including the pre-process phase (2 h), during which the initial vacuum was created, the building platform was pre-heated, and the beam was aligned, the process time (48.5 h), and the cooling down phase (6.1 h). The specific primary energy consumption of the EBM system, including pre-process and cooling phases, is 485 MJ/kg, according to the experimental characterization by Atzeni et al., 2021 [32]. Material losses during the EBM process are originated by the support volume and by the collection of non-reusable powder from the sieve and the powder recovery system (PRS), assumed to be 5% of the melted mass (parts plus supports).

In the definition of the LCA models, it was assumed a constant electricity consumption, equal to the nominal value reported in the technical sheet of each printer.

## 3. Results and discussion

### 3.1. Characterization

**Figure 2:**
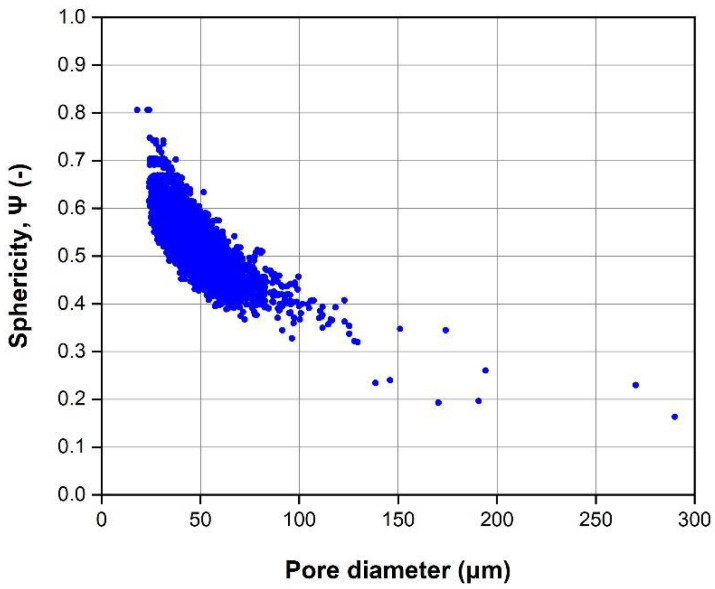
Diameter vs. sphericity of the pores.

**Figure 3:**
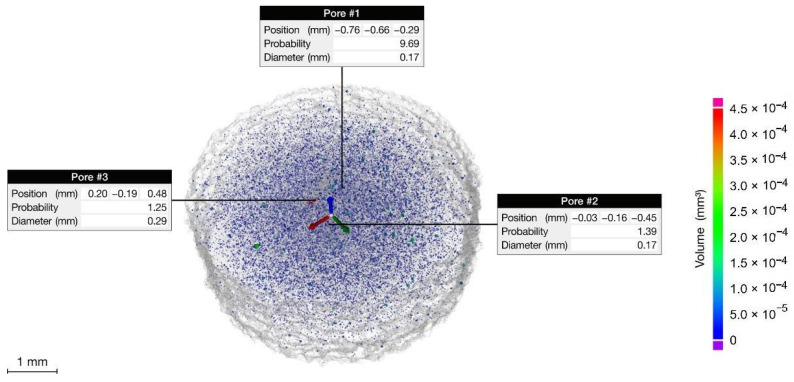
Volumetric distribution of the pores in the analyzed section.

The total porosity is 0.27%, being 80% of pores’ volume less than 0.1 × 10^−3^ mm^3^. This result agrees with the data presented in the literature (Pirozzi et al., 2019 [34]; Varney et al., 2021 [35]). Most of the pores are spherical/ellipsoidal in shape with an average diameter of 36 µm as shown in Figure 2. These pores are generated by gases released during the EBM process and are almost uniformly distributed in the analyzed volume (Figure 3). Moreover, a few large pores with irregular shape are visible, with a larger pore size of 290 µm. The origin of these pores is attributed to a lack of fusion or balling effect, that may occur in EBM due to uncontrolled variables that can affect the beam control, and consequently locally reduce the energy density. 

**Figure 4:**
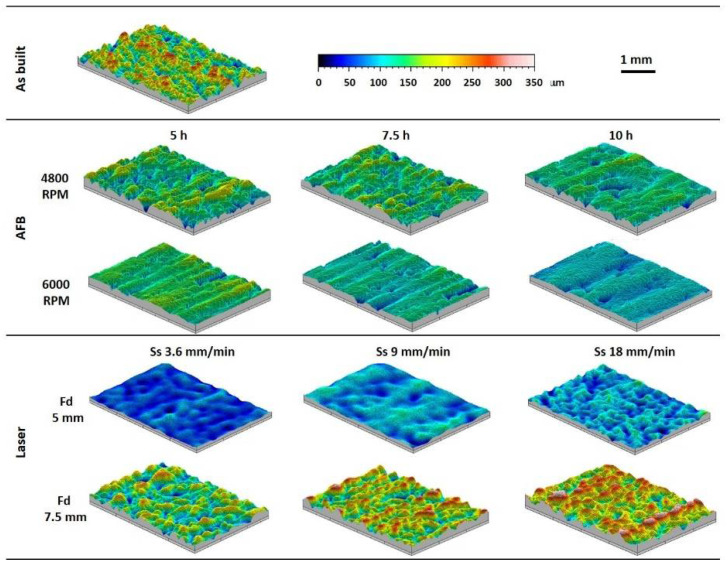
Three-dimensional maps acquired by the profiler on the as-built sample and treated ones.

The morphology of the surfaces before and after AFB and LF treatments are shown in Figure 4. It reports the comparison of the three-dimensional maps acquired by the profiler. The effect of the impact of abrasive particles and the effect of laser-surface interaction are reported for the varied process conditions. The difference between the starting morphology and the one obtained after treatments was strictly related to rotational speed and time for AFB, and to focus and speed for the laser process.

**Figure 5:**
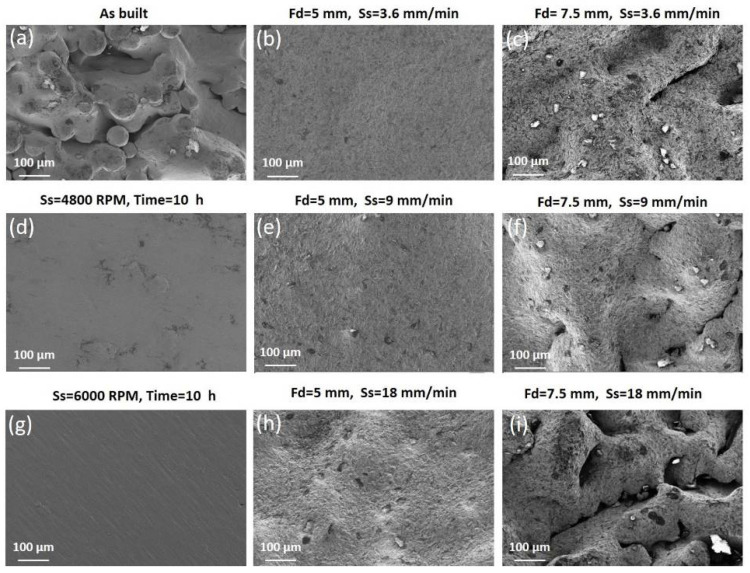
SEM analysis of the external surfaces of the as-built sample and treated ones: (**a**) As-built sample; (**b**) Laser finished sample (Fd = 5 mm, Ss = 3.6 mm/min); (**c**) Laser finished sample (Fd = 7.5 mm, Ss = 3.6 mm/min); (**d**) AFB finished sample (Ss = 4800 RPM, time = 10 h); (**e**) Laser finished sample (Fd = 5 mm, Ss = 9 mm/min); (**f**) Laser finished sample (Fd = 7.5 mm, Ss = 9 mm/min); (**g**) AFB finished sample (Ss = 6000 RPM, time = 10 h); (**h**) Laser finished sample (Fd = 5 mm, Ss = 18 mm/min); (**i**) Laser finished sample (Fd = 7.5 mm, Ss = 18 mm/min).

The effect of the process parameters on the surface topography was analyzed also by SEM. As regards AFB treatment, a smoother morphology was obtained at the higher sample rotational speed of 6000 RPM, and for increasing time (Figure 5g). As regards laser treatment, a smoother morphology was obtained for Fd = 5 mm and the lower speed (Figure 5b). The aspect of the as-built surface in Figure 5a was significantly modified after the finishing process, and the starting agglomerates were completely flattened.

**Figure 6:**
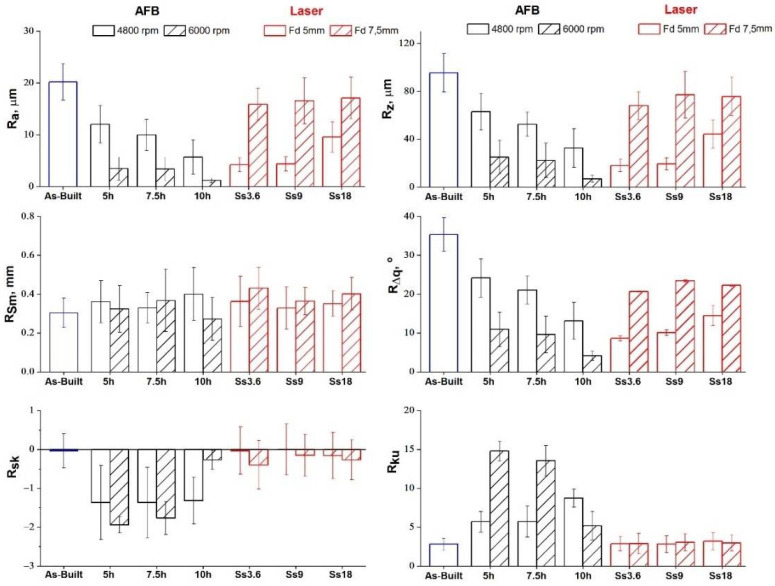
Roughness parameters of the as-built sample and treated ones for different process conditions.

To better analyze the morphology improvement according to process parameters, Figure 6 shows the trend of roughness parameters versus AFB and laser process conditions. Lower values of arithmetic mean surface roughness (Ra) and surface roughness depth (Rz) were always obtained for AFB when high-speed impacts between media and surface took place. An almost linear evolution of parameters with increasing time was found for the lower sample rotational speed. In the case of laser treatment, lower values of Ra and Rz were always obtained for Fd= 5 mm and these roughness parameters increase with increasing scan speed. The low roughness is related to the laser energy provided. At the lower focal distance (Fd = 5 mm) the amount of energy transferred to the material is enough to vaporize the higher peaks and melt the greater ones. Conversely, at a higher focal distance (Fd = 7.5 mm), the energy available to the machining is lower and the higher peaks are just melted, leaving some irregularity as explained in [28].

**Figure 7:**
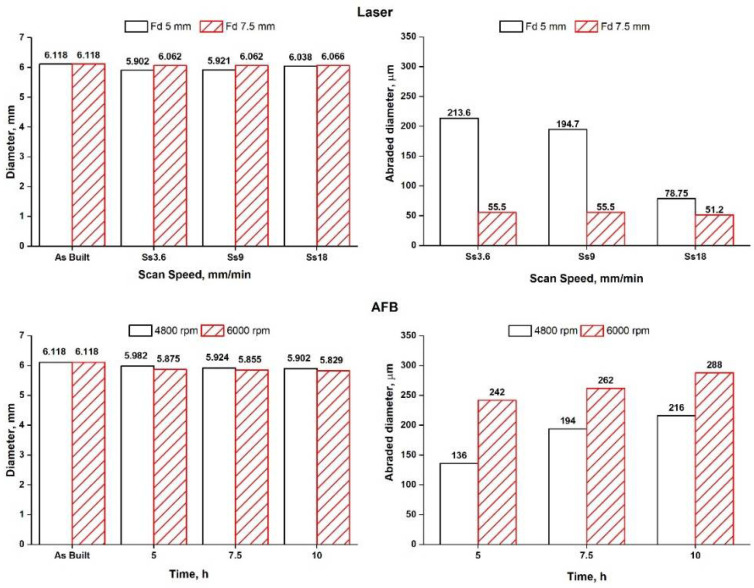
Dimensional variation of the treated samples at different process conditions.

To relate the evolution of morphology, in particular, the regression of the re-solidified metal droplets in Figure 5a by the action of abrasive or laser source, to material removal, the variation of sample dimensions was evaluated and shown in Figure 7. The variation of the diameter can be compared to Rz to understand if the removal of partially melted particles was completed during the finishing processes. Both the treatments determined the removal of semi-molten powder agglomerates, typical of additively manufactured parts.

**Figure 8:**
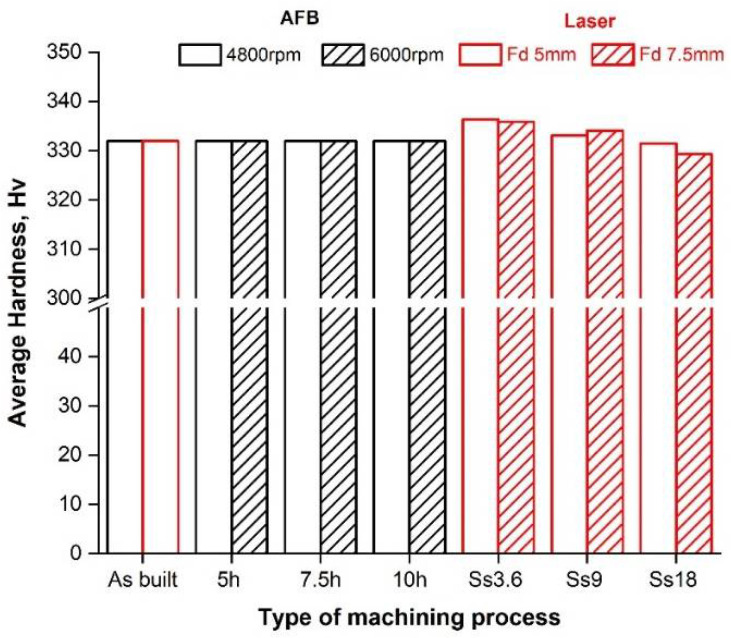
Average hardness at different process conditions.

Figure 8 reports the average microhardness analysis along the cross-sections. As regards the AFB, no trend is visible to varying conditions (the average microhardness is equal to about 330 HV for all samples, including the as-built one). Conversely, for LF it can be inferred that microhardness increases with decreasing levels of the scan speed, because of the thermal nature of the laser treatment that generally affects mechanical characteristics. Therefore, this outcome results from the melting and rapid cooling that occur during the laser finishing process. Since the thermal gradient is steeper at low scan speeds, also the change in microhardness is more obvious.

**Figure 9:**
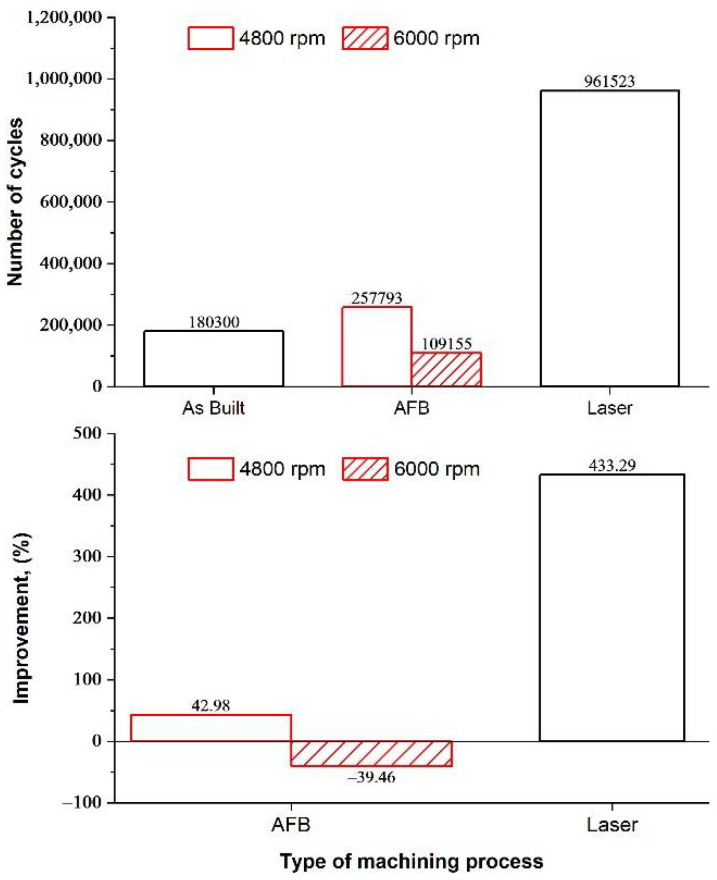
Number of cycles to failure for: as-built samples, samples treated by AFB for 10 h at different speed, laser treated samples with Fd = 5 mm and Ss = 3.6 mm/min.

The finishing processes have an effect also on the fatigue life, evaluated in this study by rotating bending tests. The number of cycles to failure of as-built and treated samples are shown in Figure 9. As known, surface and internal defects have a deleterious effect on fatigue performances, for this reason, they were analyzed and discussed. By comparing the performances of the different samples, the AFB treatment led to an increase in the fatigue life only when the lower speed was set (257,793 cycles). While the laser treatment determined a significant improvement of fatigue performances when the optimum parameters in terms of roughness (Fd = 5 mm and Ss = 3.6 mm/min) were set (961,523 cycles). The experimental results suggest that AFB and laser treatments are able to improve at the same time the finishing and fatigue behavior of additively manufactured parts, once fixed the appropriate process conditions.

### 3.2. Life Cycle Analysis

LCA models related to the two finishing technologies were defined, considering 1 sample for laser treatment and 4 samples for AFB, so as to have a comparable number of cycles to fatigue failure. All the phases of finishing were considered: on the one hand AFB treatment and cleaning in ultrasonic bath, on the other laser treatment, sandblasted, and cleaning in an ultrasonic bath. Both materials (raw material, support material, chemical agents, etc.) and energy (compressed air, electricity, etc.) were considered. 

The cumulative energy demand and the mass flow to produce each sample are reported in Table 2.

**Figure 10:**
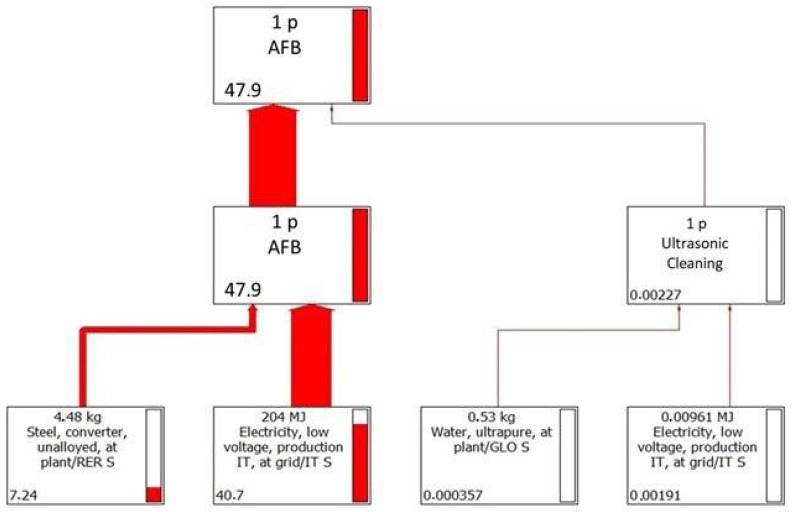
Life cycle of AFB process.

**Figure 11:**
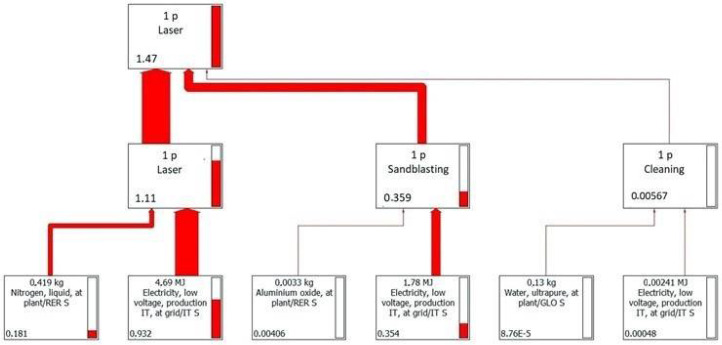
Life cycle of laser process.

The input/output data of each finishing process are listed in Table 3. From Table 4, it can be noticed that fluidized bed processing has a higher environmental impact in terms of global warming potential (GWP) than laser. This impact originates almost totally from the fluidized bed treatment, while the ultrasonic bath is practically negligible. Additionally in the LF, the ultrasonic bath is negligible and almost all of the impact of the processing is due to the contribution of the laser treatment, even if sandblasting cannot be considered a negligible process. Besides, the entire process is represented through a tree diagram where both the various sub-areas and the consumption of resources and energy are reported to individuate the critical flow in terms of CO_2_ equivalent. In Figure 10, the graph shows that the greatest contribution of CO_2_ equivalent is given by the electricity used in AFB. However, unlike the contributions given by water and electricity in the ultrasonic bath, the contribution made by the steel grit is not completely negligible. From Figure 11, it can be concluded that electricity is the most influential contribution of LF, but the impact due to the nitrogen used to avoid the oxidation of the material cannot be underestimated.

## 4. Conclusions

Two finishing techniques, namely AFB and LF, were carried out on Ti-6Al-4V parts fabricated by EBM to compare their effects on fatigue life. Based on preliminary investigations on the effect of process parameters, an extensive analysis of both mechanical properties and fatigue performances is provided. The internal porosity was evaluated by means of micro-CT scanning and image analysis. The morphology of the finished surfaces was analyzed by SEM and profilometry. Surface hardness was obtained through a micro-scale tester. Fatigue tests were performed using a rotating bending machine. Then, a comparative life cycle assessment was carried out to determine the environmental impact of these finishing technologies. The main results can be summarized as follows:

- The porosity of the EBM parts is 0.27%. Most of the pores are uniformly distributed spherical/ellipsoidal pores generated by the gas released during the EBM process. Few irregular large pores are originated by the lack of fusion that may occur because of the uncontrolled variables of the EBM process.
- Both the finishing processes determine the removal of the typical semi-molten powder agglomerates. In AFB, a smoother morphology is obtained at the higher sample rotational speed of 6000 RPM and surface roughness increases with time. In LF, a smoother morphology is obtained for Fd = 5 mm at the lower speed.
- The average microhardness is not affected by the process conditions of AFB (about 330 HV for all samples, including the as-built one). As regards the LF, the microhardness increases with decreasing levels of the scan speed.
- The fatigue life is affected by the finishing processes. AFB treatment leads to an increase in the fatigue life when the lower speed is set (257,793 cycles). While the LF improves fatigue performances when Fd = 5 mm and Ss = 3.6 mm/min are set (961,523 cycles).
- LF has a very low environmental impact (1.474) compared to ABF (47.920) in terms of IPCC GWP 100° metrics. In LF, sandblasting is a significant contribution, but the most influent contribution is given by the electricity used. The use of the abrasive powder can be neglected, just as with the ultrasonic bath.

## Figures and Tables

**Table 1:** Main characteristics of Arcam A2X system.

Characteristic	Value	Unit
maximum build size	200 × 200 × 380	mm^3^
beam power	3000	W
cathode	Tungsten filament	-
minimum beam diameter	250	µm
maximum beam translation speed	800	m/s
vacuum base pressure	5 × 10^−4^	mbar
build atmosphere (partial pressure of He)	2 × 10^−3^	mbar
He consumption	1	l/h
CAD interface standard	Standard triangulation language	-

**Table 2:** Cumulative Energy Demand and mass flow per sample.

Cumulative Energy Demand (CED)	Value	Unit
powder production	69.1	MJ
EBM	52.2	MJ
**Mass Flow**	**Value**	**Unit**
mass part	0.098	kg
mass losses, EBM	0.010	kg
mass losses, atomization	0.003	kg

**Table 3:** Input/output consumption of the analyzed finishing processes.

AFB Finishing Input/Output	Consumption	Unit
AFB treatment, abrasive material	4.48	kg
electricity (blower, spindle)	56.8	kWh
water (ultrasonic bath)	0.53	l
electricity (ultrasonic cleaning system)	2.67	Wh
**Laser Finishing Input/Output**	**Consumption**	**Unit**
gas (nitrogen)	0.419	kg
electricity (laser, handling motors)	1.302	kWh
corundum	3.33 × 10^−3^	kg
electricity (sandblasting compressor)	0.495	kWh
water (ultrasonic bath)	0.13	L
electricity (ultrasonic cleaning system)	0.67	Wh

**Table 4:** Computed values of IPCC GWP 100° for each analyzed finishing process.

AFB Finishing IPCC GWP 100°	Value	Unit
AFB treatment	47.918	kg CO_2_ eq
ultrasonic bath	0.002	kg CO_2_ eq
total	47.920	kg CO_2_ eq
**Laser Finishing IPCC GWP 100°**	**Value**	**Unit**
laser treatment	1.114	kg CO_2_ eq
sandblasting	0.359	kg CO_2_ eq
ultrasonic bath	0.001	kg CO_2_ eq
total	1.474	kgCO_2_ eq

## Data Availability

The data are not publicly available due to information used for another publication.

## References

[1] Levy G.N., Schindel R., Kruth J.P. (2003). Rapid Manufacturing and Rapid Tooling with Layer Manufacturing (LM) Technologies, State of the Art and Future Perspectives. CIRP Ann. Manuf. Technol..

[2] Tagliaferri V., Trovalusci F., Guarino S., Venettacci S. (2019). Environmental and Economic Analysis of FDM, SLS and MJF Additive Manufacturing Technologies. Materials.

[3] Qian M., Bourell D.L. (2017). Additive Manufacturing of Titanium Alloys. JOM.

[4] Froes F.H. (2018). Additive Manufacturing of Titanium Components: An Up-Date. Met. Powder Rep..

[5] Wycisk E., Solbach A., Siddique S., Herzog D., Walther F., Emmelmann C. (2014). Effects of Defects in Laser Additive Manufactured Ti-6Al-4V on Fatigue Properties. Phys. Proced..

[6] Zhang L.C., Liu Y., Li S., Hao Y. (2018). Additive Manufacturing of Titanium Alloys by Electron Beam Melting: A Review. Adv. Eng. Mater..

[7] Koike M., Greer P., Owen K., Lilly G., Murr L.E., Gaytan S.M., Martinez E., Okabe T. (2011). Evaluation of Titanium Alloys Fabricated Using Rapid Prototyping Technologies-Electron Beam Melting and Laser Beam Melting. Materials.

[8] Murr L.E., Gaytan S.M., Ceylan A., Martinez E., Martinez J.L., Hernandez D.H., Machado B.I., Ramirez D.A., Medina F., Collins S. (2010). Characterization of Titanium Aluminide Alloy Components Fabricated by Additive Manufacturing Using Electron Beam Melting. Acta Mater..

[9] Seifi M., Salem A., Satko D., Shaffer J., Lewandowski J.J. (2017). Defect Distribution and Microstructure Heterogeneity Effects on Fracture Resistance and Fatigue Behavior of EBM Ti-6Al-4V. Int. J. Fatigue.

[10] Seifi M., Dahar M., Aman R., Harrysson O., Beuth J., Lewandowski J.J. (2015). Evaluation of Orientation Dependence of Fracture Toughness and Fatigue Crack Propagation Behavior of As-Deposited ARCAM EBM Ti-6Al-4V. JOM.

[11] Gorelik M. (2017). Additive Manufacturing in the Context of Structural Integrity. Int. J. Fatigue.

[12] Tan X., Kok Y., Tan Y.J., Vastola G., Pei Q.X., Zhang G., Zhang Y.W., Tor S.B., Leong K.F., Chua C.K. (2015). An Experimental and Simulation Study on Build Thickness Dependent Microstructure for Electron Beam Melted Ti-6Al-4V. J. Alloys Compd..

[13] Chern A.H., Nandwana P., Yuan T., Kirka M.M., Dehoff R.R., Liaw P.K., Duty C.E. (2019). A Review on the Fatigue Behavior of Ti-6Al-4V Fabricated by Electron Beam Melting Additive Manufacturing. Int. J. Fatigue.

[14] Beretta S., Romano S. (2017). A Comparison of Fatigue Strength Sensitivity to Defects for Materials Manufactured by AM or Traditional Processes. Int. J. Fatigue.

[15] Fatemi A., Molaei R., Sharifimehr S., Phan N., Shamsaei N. (2017). Multiaxial Fatigue Behavior of Wrought and Additive Manufactured Ti-6Al-4V Including Surface Finish Effect. Int. J. Fatigue.

[16] Schijve J. (2003). Fatigue of Structures and Materials in the 20th Century and the State of the Art. Int. J. Fatigue.

[17] Vastola G., Zhang G., Pei Q.X., Zhang Y.W. (2015). Modeling and Control of Remelting in High-Energy Beam Additive Manufacturing. Addit. Manuf..

[18] Hrabe N., Quinn T. (2013). Effects of Processing on Microstructure and Mechanical Properties of a Titanium Alloy (Ti-6Al-4V) Fabricated Using Electron Beam Melting (EBM), Part 1: Distance from Build Plate and Part Size. Mater. Sci. Eng. A.

[19] Hrabe N., Quinn T. (2013). Effects of Processing on Microstructure and Mechanical Properties of a Titanium Alloy (Ti-6Al-4V) Fabricated Using Electron Beam Melting (EBM), Part 2: Energy Input, Orientation, and Location. Mater. Sci. Eng. A.

[20] Małecka J., Rozumek D. (2020). Metallographic and Mechanical Research of the O-Ti2AlNb Alloy. Materials.

[21] Chan K.S. (2015). Characterization and Analysis of Surface Notches on Ti-Alloy Plates Fabricated by Additive Manufacturing Techniques. Surf. Topogr. Metrol. Prop..

[22] Bagehorn S., Wehr J., Maier H.J. (2017). Application of Mechanical Surface Finishing Processes for Roughness Reduction and Fatigue Improvement of Additively Manufactured Ti-6Al-4V Parts. Int. J. Fatigue.

[23] Ahmed N., Abdo B.M., Darwish S., Moiduddin K., Pervaiz S., Alahmari A.M., Naveed M. (2017). Electron Beam Melting of Titanium Alloy and Surface Finish Improvement through Rotary Ultrasonic Machining. Int. J. Adv. Manuf. Technol..

[24] Yang G., Wang B., Tawfiq K., Wei H., Zhou S., Chen G. (2017). Electropolishing of Surfaces: Theory and Applications. Surf. Eng..

[25] Piotrowski O., Madore C., Landolt D. (1998). Electropolishing of Titanium and Titanium Alloys in Perchlorate-Free Electrolytes. Plat. Surf. Finish..

[26] Ukar E., Lamikiz A., Martínez S., Tabernero I., Lacalle L.N.L.D. (2012). Roughness Prediction on Laser Polished Surfaces. J. Mater. Process. Technol..

[27] Barletta M. (2009). Progress in Abrasive Fluidized Bed Machining. J. Mater. Process. Technol..

[28] Genna S., Rubino G. (2020). Laser Finishing of Ti6Al4V Additive Manufactured Parts by Electron Beam Melting. Appl. Sci..

[29] Atzeni E., Rubino G., Salmi A., Trovalusci F. (2020). Abrasive Fluidized Bed Finishing to Improve the Fatigue Behaviour of Ti6Al4V Parts Fabricated by Electron Beam Melting. Int. J. Adv. Manuf. Technol..

[30] Nicoletto G., Konečná R., Frkáň M., Riva E. (2018). Surface Roughness and Directional Fatigue Behavior of As-Built EBM and DMLS Ti6Al4V. Int. J. Fatigue.

[31] du Plessis A., Sperling P., Beerlink A., Tshabalala L., Hoosain S., Mathe N., le Roux S.G. (2018). Standard Method for MicroCT-Based Additive Manufacturing Quality Control 1: Porosity Analysis. MethodsX.

[32] Atzeni E., Catalano A.R., Priarone P.C., Salmi A. (2021). The Technology, Economy, and Environmental Sustainability of Isotropic Superfinishing Applied to Electron-Beam Melted Ti-6Al-4V Components. Int. J. Adv. Manuf. Technol..

[33] Paris H., Mokhtarian H., Coatanéa E., Museau M., Ituarte I.F. (2016). Comparative Environmental Impacts of Additive and Subtractive Manufacturing Technologies. CIRP Ann. Manuf. Technol..

[34] Pirozzi C., Franchitti S., Borrelli R., Diodati G., Vattasso G. (2019). Experimental Study on the Porosity of Electron Beam Melting-Manufactured Ti6Al4V. J. Mater. Eng. Perform..

[35] Varney T.C., Quammen R.N., Telesz N., Balk T.J., Wessman A., Rottmann P.F. (2021). Effects of Pore Geometry on the Fatigue Properties of Electron Beam Melted Titanium-6Al-4V. Metall. Mater. Trans. A Phys. Metall. Mater. Sci..

